# Supplementary material for: Endo-Exoglucanase Synergism for Cellulose Nanofibril Production Assessment and Characterization
Source: Molecules. 2023 Jan 18;28(3):948. doi: 10.3390/molecules28030948 (PMC9921176; doi:10.3390/molecules28030948)
Supplement: Supplementary file 1 [file molecules-28-00948-s001.zip › molecules-2102443-supplementary.pdf]

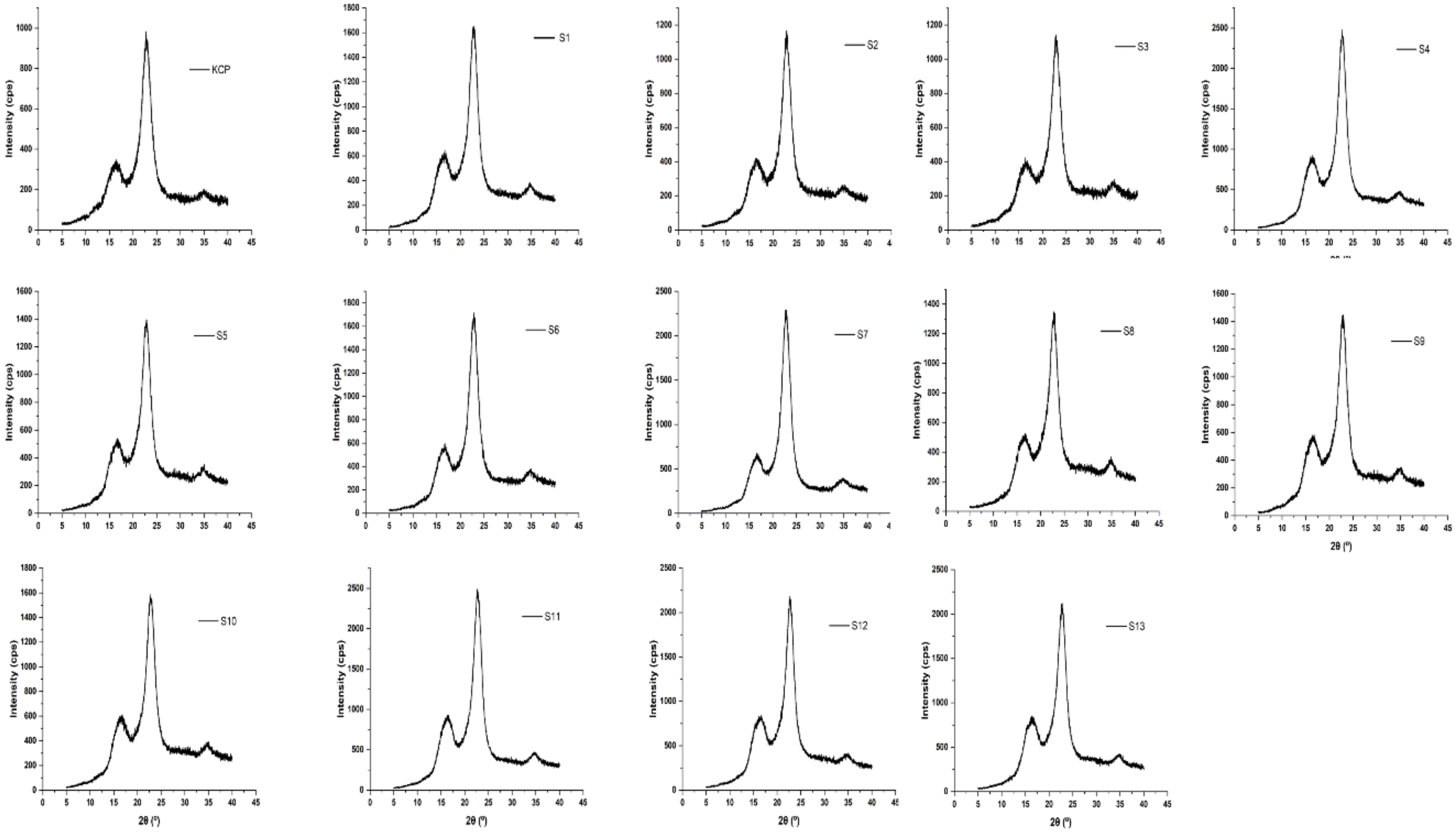

Figure S1. Diffractograms of KCP and CNF samples produced by enzymatic hydrolysis

Model S1: Determination of Optimal Model of Enzyme Mixtures

The mathematical model that best described the minimum glucose release after enzymatic hydrolysis of KCP was analyzed using Statistica® software. The mixture design of the simplex-centroid approach was used with the help of Design-Experts to optimize the enzymatic cocktail with three cellulase enzymes for CNF production (A: EGU, B: ExG, C: BG). The evaluated response variable is Y: minimal glucose released (g/L). The full quartic model was chosen because it was the one that presented the highest coefficient of determination (R^2), equivalent to 86.03 %. In contrast, the mathematical models of complete cubic, quadratic, and linear showed R^2 values of 78.21 %, 50.62 % and 21.05 %, respectively. The full quartic model obtained is displayed as follows:

$$Y = 0.039 \cdot A + 0.033 \cdot B + 0.038 \cdot C - 0.060 \cdot AB - 0.044 \cdot AC - 0.24 \cdot AB(A - B) + 1.18 \cdot ABCC - 0.46 \cdot AB(A - B)^2$$

Statistically, the model explains the response based on the response Y significant p-value in the quartic model. A factor was regarded to have a significant effect on CNF enzymatic synthesis if its value was less than 0.05 (95 % confidence level). The value was obtained from Fisher's distribution table (Table S1), which depends on the error degree of freedom (DF) and the mean squares (MS). Five of the fifteen effects and interactions presented were not statistically significant. BC and AC(A-C) had p-values greater than 0.05 and small Fisher's statistics; they are non-significant interactions, so they were discarded from the full quartic model to obtain the previous equation. The optimized response of the centroid simplex mixture design coincides with the interactions of the full quartic model that presented the highest Fisher's statistic values (smallest p-values), AB and AB(A-B), prevailing their significance in the model.

Table S1. Estimated regression coefficients for the full quartic model.

Effect/Interaction	Coefficient	Fisher Statistical	p-value
A	0.039	1.16	$\ll 0.05$
B	0.033	1.16	$\ll 0.05$
C	0.038	1.16	$\ll 0.05$
AB	−0.060	16.90	0.002
AC	−0.044	9.09	0.013
BC	−0.0060	0.17	0.690
AB(A-B)	-0.24	16.96	0.002
AC(A-C)	0.014	0.05	0.833
BC(B-C)		was not statistically significant	
AABC		was not statistically significant	
ABBC		was not statistically significant	
ABCC	1.18	14.65	0.003
$AB(A-B)^2$	−0.46	12.76	0.005
$AC(A-C)^2$		was not statistically significant	
$BC(B-C)^2$		was not statistically significant	

BC and AC(A-C) had p-values greater than 0.05 and small Fisher's statistics; they are non-significant interactions.
